# Retraction: Comparison of central versus peripheral delivery of pregabalin in neuropathic pain states

**DOI:** 10.1186/1744-8069-10-20

**Published:** 2014-04-02

**Authors:** Jose A Martinez, Manami Kasamatsu, Alma Rosales-Hernandez, Leah R Hanson, William H Frey, Cory C Toth

**Affiliations:** 1 Department of Clinical Neurosciences and the University of Calgary, Calgary, AB, Canada; 2 Alzheimer's Research Center, Regions Hospital, and HealthPartners Research Foundation, St. Paul, MN, USA; 3 Department of Pharmaceutics, University of Minnesota, Minneapolis, MN, USA

## Retraction

The corresponding author Cory C Toth would like to retract the article [1]. It has come to light that the data obtained at the University of Calgary presented in Figure 4A and Figure 5 have been manipulated which was unrecognized by the corresponding author. The University of Calgary has investigated this case and supports the decision to retract the article. We apologize for misleading the readership of *Molecular Pain*.
